# Manifestation of a sellar hemangioblastoma due to pituitary apoplexy: a case report

**DOI:** 10.1186/1752-1947-5-496

**Published:** 2011-10-04

**Authors:** Ralph T Schär, Istvan Vajtai, Rahel Sahli, Rolf W Seiler

**Affiliations:** 1 Department of Neurosurgery, Inselspital, University Hospital Bern, 3010 Bern, Switzerland; 2 Section of Neuropathology, Institute of Pathology, University of Bern, 3010 Bern, Switzerland; 3 Division of Endocrinology, Diabetes and Clinical Nutrition, Inselspital, University Hospital Bern, 3010 Bern, Switzerland

## Abstract

**Introduction:**

Hemangioblastomas are rare, benign tumors occurring in any part of the nervous system. Most are found as sporadic tumors in the cerebellum or spinal cord. However, these neoplasms are also associated with von Hippel-Lindau disease. We report a rare case of a sporadic sellar hemangioblastoma that became symptomatic due to pituitary apoplexy.

**Case presentation:**

An 80-year-old, otherwise healthy Caucasian woman presented to our facility with severe headache attacks, hypocortisolism and blurred vision. A magnetic resonance imaging scan showed an acute hemorrhage of a known, stable and asymptomatic sellar mass lesion with chiasmatic compression accounting for our patient's acute visual impairment. The tumor was resected by a transnasal, transsphenoidal approach and histological examination revealed a capillary hemangioblastoma (World Health Organization grade I). Our patient recovered well and substitutional therapy was started for panhypopituitarism. A follow-up magnetic resonance imaging scan performed 16 months postoperatively showed good chiasmatic decompression with no tumor recurrence.

**Conclusions:**

A review of the literature confirmed supratentorial locations of hemangioblastomas to be very unusual, especially within the sellar region. However, intrasellar hemangioblastoma must be considered in the differential diagnosis of pituitary apoplexy.

## Introduction

Hemangioblastomas (HBLs) are benign, slowly growing and highly vascular tumors of the central nervous system (CNS), accounting for just 1% to 2.5% of all intracranial neoplasms, and 7% to 12% of primary tumors located in the posterior fossa [1]. In up to one in four cases of HBL there is an association with von Hippel-Lindau (VHL) disease [2], a rare autosomal dominant condition that predisposes patients to multisystemic neoplastic disorders such as HBLs of the CNS, retinal angiomas, renal cell carcinoma, pheochromocytomas, serous cystadenomas and neuroendocrine tumors of the pancreas. VHL-associated HBLs tend to occur in younger patients and are often multiple in occurrence [2-4]. Sporadic HBLs, however, are mostly solitary lesions and predominantly found within the cerebellum or spinal cord. Supratentorial HBLs, which are more often associated with VHL disease [3,4], are a rare entity with just over 100 reported cases to date [5]. HBLs originating from the sellar or suprasellar region are exceptional, especially in cases with no association with VHL disease.

We report here what is, to the best of our knowledge, the seventh sporadic case in the literature of sellar HBL, which presented with pituitary apoplexy. We also review the literature on cases of HBL within the sellar and suprasellar region.

## Case presentation

**Figure 1:**
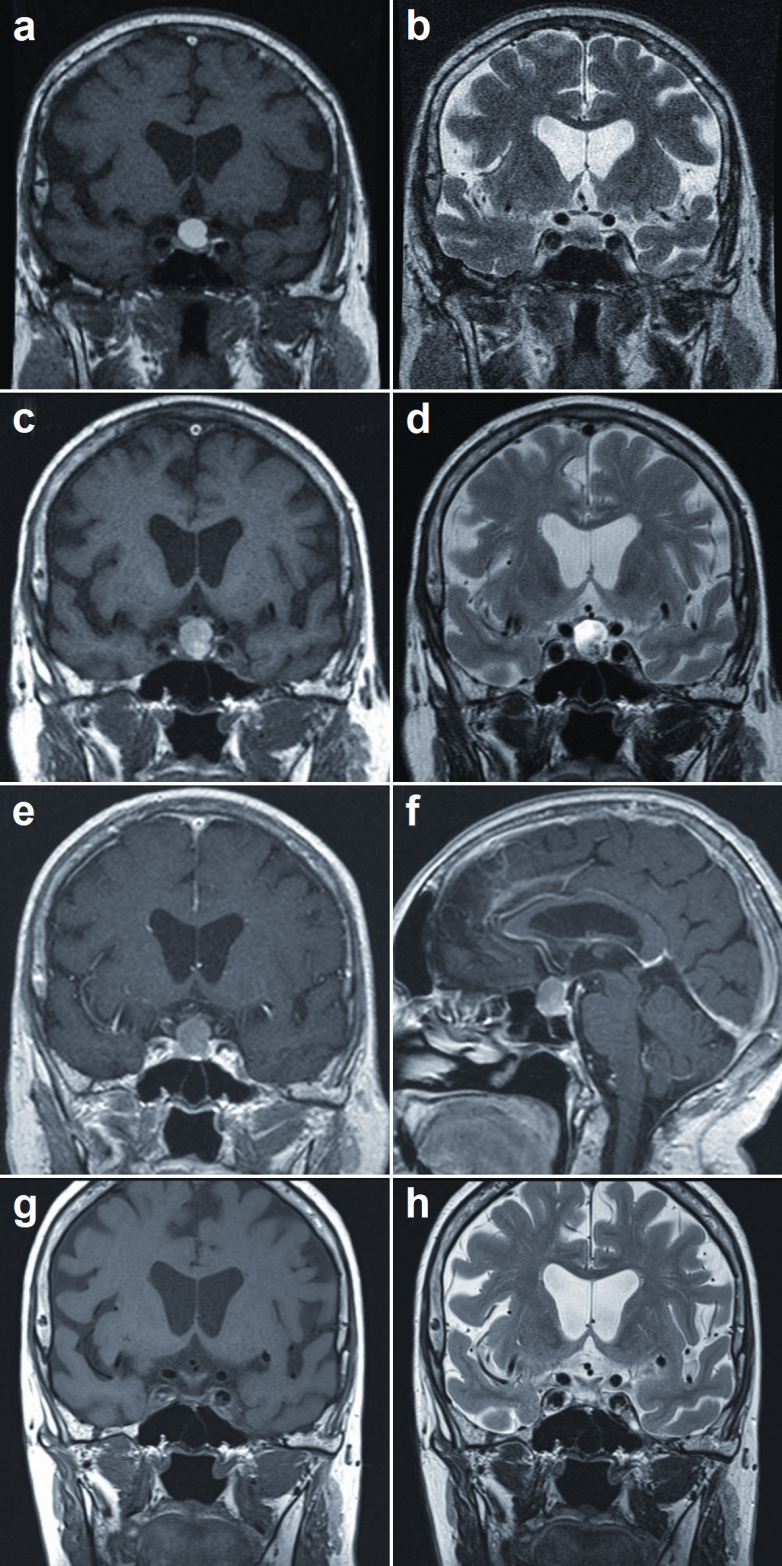
**MRI images of patient's brain**. **(A, B) **T1- and T2- weighted MRI scans taken two years prior to current presentation. **(C) **T1-weighted MRI scan of patient's brain, revealing a partly vesicular hyperintense, and slightly increased (compared to A and B) intrasellar and suprasellar mass of 16 mm in diameter, with progressive compression of the prechiasmatic portions of her optic nerves bilaterally. **(D) **T2-weighted MRI scan showing the vesicular portion as hypointense; normal pituitary tissue could not be clearly delineated. **(E, F) **There was no evident enhancement on T1-weighted imaging after intravenous administration of gadolinium. **(G, H) **An MRI scan taken 16 months postoperatively showed regular display of the remaining pituitary gland with good chiasmatic decompression and no signs of tumor recurrence.

An 80-year-old Caucasian woman was admitted to our hospital with a 12-year history of an endocrine inactive steady sellar mass lesion (13 mm in diameter; Figure 1A, B). Our patient had been previously asymptomatic with no pituitary hormone deficiency or visual impairments. Moreover, our patient had a medical history of good health with only minor health issues that included hypertension and osteoporosis. However, prior to hospital admission, she had recently experienced two severe headache attacks; the last episode was accompanied by nausea, vomiting and blurred vision. Hyponatremia (120 mEq/L) with low serum osmolality (247 mOsm/L) and highly elevated urine osmolality (695 mOsm/L) were detected. An endocrinological investigation revealed hypocortisolism with no other hormone disturbances. Fundoscopy showed no pathological findings. However, further ophthalmologic examination with Goldman perimetry confirmed a bitemporal hemianopsia accentuated on her right side. Her neurological examination results were otherwise normal. After substitution therapy with hydrocortisone, our patient rapidly improved and her headaches subsided.

Findings from a magnetic resonance imaging (MRI) scan were suggestive of an acute hemorrhage of the sellar process, consistent with pituitary apoplexy (Figure 1C-F). Except for an age-consistent vascular leukoencephalopathy, the diagnostic imaging showed no further pathological findings. Our tentative diagnosis at this point was a pituitary adenoma with pituitary apoplexy.

Due to these clinical and radiological findings, the decision was made to surgically remove the tumor. A gross total extirpation using a transnasal, transsphenoidal approach to the pituitary mass was successfully performed. Intraoperatively, the tumor appeared yellowish-brown, was relatively firm and was located within a sellar hematoma cavity, which was evacuated.

Postoperatively, our patient's visual field deficits improved markedly on clinical examination and Goldman perimetry confirmed a partial recovery of her bitemporal visual field deficits. Endocrinological studies showed panhypopituitarism with partial and transient diabetes insipidus. Our patient received substitution therapy with hydrocortisone, levothyroxine and transient therapy with desmopressin. Overall, our patient remained in good health with a satisfactory level of performance. A repeat MRI scan taken 16 months after surgery showed good chiasmatic decompression with no residual tumor mass (Figure 1G, H).

**Figure 2:**
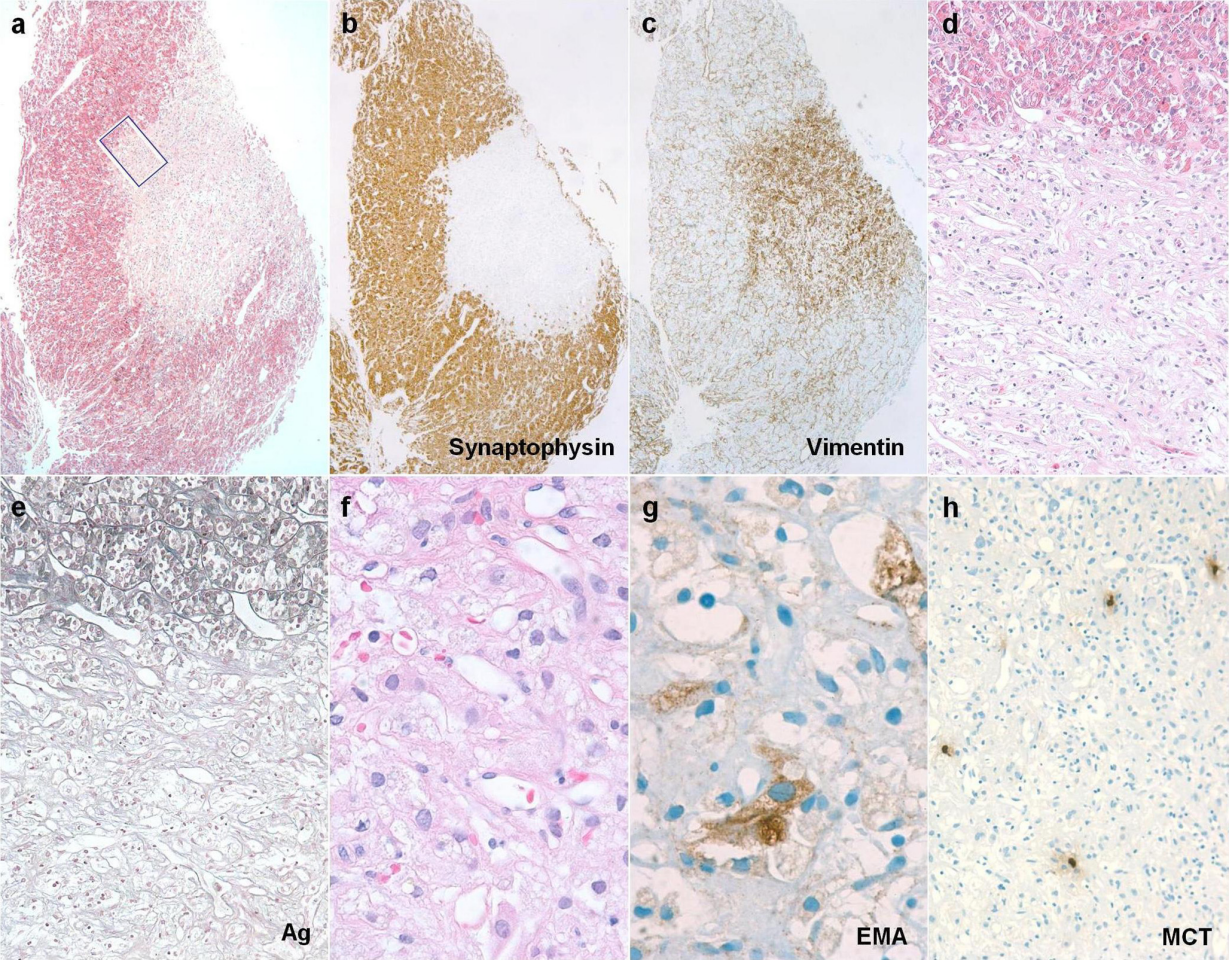
**Overview showing well circumscribed HBL nodule partly surrounded by a crescent-shaped mantle of peritumoral pituitary parenchyma**. (A) Optical contrast between the faint eosinophilic hue of the HBL nidus and bright red granular quality of adjacent somatotrophs. (B, C) Adjacent section planes treated with immunohistochemistry, showing segregation of adenohypophyseal neuroendocrine cells (B) and mesenchymal-like immunophenotype (C) of the HBL nodule. (D) Detail view of boxed area in (A) shows the HBL to be comprised of an irregular reticular meshwork of tortuous, thin-walled capillaries that tend to be interspersed with pale stromal cells. (E) Gomori's reticulin stain highlighting the brisk transition from the acinar outline of native adenohypophyseal follicles (upper third) to the vascular-dominated basement membrane pattern of HBL. (F) High-power view of HBL showing polygonal contours and cytoplasmic vacuolation of stromal cells encased by capillaries. Some nuclear pleomorphism, as also evident in this microscopic field, is of no prognostic significance. (G) A minority of stromal cells were stained for epithelial membrane antigen. (H) Scattered mast cells are a characteristic complement of HBL. If not labeled otherwise, microphotographs have been made using hematoxylin and eosin stain. Original magnifications: (A-C) × 30; (D, E, H) × 100; (F, G) × 400.

The resected tumor was examined with light microscopy, which revealed a small, well circumscribed, non-adenomatous tumor surrounded by slightly compressed remnants of adenohypophyseal parenchyma (Figure 2A-C). The tumor was richly vascularized with an observable reticular mesh of thin-walled capillaries interspersed with large epithelioid-looking cells (Figure 2D, E). Pale eosinophilic cytoplasm showed xanthomatous or vacuolar change (Figure 2F). Immunohistochemistry confirmed the expression of the endothelial-associated markers CD31 and CD34 in the intratumoral capillaries, although not in the stromal cells themselves. Conversely, the stromal cells were diffusely immunoreactive for vimentin, with a minority of cells also coexpressing S100 protein and epithelial membrane antigen (Figure 2G). No inflammatory infiltrate was detected except for the occasional mast cell (Figure 2H). Staining for cytokeratins tested negative, as did the Langerhans-cell-associated marker CD1a. Less than 1% of lesional cell nuclei were labeled with the cell proliferation-associated antigen Ki-67.

Given the above findings, we identified the tumor as an intrapituitary example of capillary hemangioblastoma (World Health Organization grade I). Since our patient displayed no clinical stigmata of VHL disease, genetic testing was not performed.

## Discussion

Based on previous studies, the occurrence of supratentorial HBLs is thought to be in the range of 2% to 8% of all HBLs [3,4,6], accounting for 116 reported cases from 1902 to 2004 [5]. Supratentorial tumors were mostly found in the frontal, parietal or temporal lobes [7].

No more than 27 reported cases to date (including our patient's case) describe HBLs originating in the sellar and suprasellar region (see [1] and references therein, and [2,8-11]) of which 18 were confirmed with histopathology (Table 1). Of the 27 cases, only seven (26%) were sporadic. In accordance with previous studies, the average age at presentation of patients with sporadic HBLs (52.4 years) was greater than patients affected with the VHL syndrome (35.8 years), excluding two cases with postmortem diagnosis (Table 1, cases 1 and 2) and one case not stating VHL association [10].

**Table 1:** Literature review of reported cases of HBL confirmed by histopathology in the sellar region

Case	Reference	Age (years), sex	Symptoms	Location	VHL	Surgery for sellar HBL	Follow-up
1	[15]	84, M	None	Intrasellar (anterior lobe)	Yes	None, autoptic finding	NA

2	[16]	26, M	Blurred vision, headache, ataxia	Intrasellar (anterior lobe)	Yes	None, autoptic finding	NA

3	[17]	19, M	Nausea, vertigo, ataxia	Suprasellar	Yes	Total resection	NA

4	[18]	19, F	Headache, amenorrhea-galactorrhea	Pituitary stalk	No	Total resection	Panhypopituitarism

5	[2]	35, F	Headache, amenorrhea, diabetes insipidus	Pituitary stalk	No	Yes, details NA	NA

6	[9]	60, F	Partial hemianopsia	Suprasellar	Yes	None, gamma knife radiosurgery	Syndrome of inappropriate secretion of antidiuretic hormone at 22-month follow-up

7	[19]	11, F	Headache, bitemporal hemianopsia, adrenocorticotropic hormone and growth hormone deficiency	Intrasellar	Yes	Subtotal resection and adjuvant radiosurgery	Headache improved, no residual tumor, panhypopituitarism

8	[20]	57, F	Diplopia, sixth nerve palsy	Intrasellar and sphenoid sinus	No	Subtotal resection	Partial improvement of sixth nerve palsy

9	[21]	20, F	Panhypopituitarism, diabetes insipidus	Suprasellar and pituitary stalk	Yes	Total resection	Stable panhypopituitarism, no residual tumor at 53-month follow-up

10	[22]	33, F	Irregular menses	Pituitary stalk	Yes	Subtotal resection	No neurological deficits or pituitary dysfunction, stable residual tumor at six-month follow-up

11	[23]	62, M	Visual disturbance	Suprasellar	No	Total resection	NA

12	[24]	60, M	Bitemporal hemianopsia, panhypopituitarism	Intrasellar and suprasellar	No	Transsphenoidal biopsy	NA

13	[25]	40, F	Oligomenorrhea, cognitive impairment	Intrasellar and suprasellar	Yes	Subtotal resection and gamma knife radiosurgery	NA

14	[26]	54, M	Headache, visual loss	Suprasellar	No	Total resection	Partial improvement of visual loss, no tumor recurrence at five-year follow-up

15	[26]	38, M	Headache, visual loss	Suprasellar	Yes	Subtotal resection	NA

16	[1]	51, F	Blurred vision	Pituitary stalk	Yes	Total resection	Panhypopituitarism, visual acuity improved

17	[27]	59, F	Fatigue, visual loss	Suprasellar	NS	Total resection	Panhypopituitarism, no tumor recurrence at three-year follow-up

18	Present case	80, F	Headache, bitemporal hemianopsia, hypocortisolism	Intrasellar	No	Total resection	Headache subsided, visual field deficits improved, panhypopituitarism, no tumor recurrence at 16-month follow-up

F: female patient; M: male patient; NA: not available.

While information on clinical features is derived from reports of sellar and suprasellar HBLs causing symptoms generally related to mass effect, a long presymptomatic stage can be assumed. Of a total of 250 patients with VHL disease enrolled in a prospective study, eight incidentally discovered HBLs located in the pituitary stalk remained stable during a mean follow-up of 41.4 ± 14 months [8]. Also, in our patient's case, the sellar lesion, initially diagnosed as an incidental finding on MRI performed for an unrelated reason, remained stable for 12 years.

Overall, the unexpected nature and the unspecific presentation render an accurate preoperative diagnosis of sporadic HBLs challenging. In our patient, the apoplexy of a well known sellar mass suggested a pituitary macroadenoma; clinical apoplexy was observed in 0.6% to 9.0% of these cases [12]. The typical, albeit not pathognomonic, radiological feature of HBLs is that they can be identified as an enhancing lesion on T1-weighted MRI scans. This finding was lacking in our case due to acute hemorrhage of the lesion.

The main histological differential diagnosis of HBL, irrespective of location, is metastatic clear cell carcinoma. In our patient, lack of immunoreactivity for cytokeratins along with a negligibly low proliferation index allowed for this alternative to be confidently ruled out. In the peculiar context of intrapituitary occurrence, we also addressed the possibility of xanthomatous hypophysitis and Langerhans cell histiocytosis [13,14]. The non-inflammatory character of the lesion in our case strongly argued against xanthomatous hypophysitis (or sellar xanthogranuloma). However, the circumscribed rather than infiltrative pattern of this solitary intrapituitary nodule, one devoid of CD1a immunoreactivity, was an intuitive obstacle against seriously considering Langerhans cell histiocytosis.

## Conclusions

Supratentorial HBLs are rare, especially within the sellar region and without an association with VHL disease. However, our patient's case shows that intrasellar HBL must be considered in the differential diagnosis of pituitary apoplexy.

## Consent

Written informed consent was obtained from the patient for publication of this case report and any accompanying images. A copy of the written consent is available for review by the Editor-in-Chief of this journal.

## Competing interests

The authors declare that they have no competing interests.

## Authors' contributions

RTS was responsible for the conception and drafting of the manuscript, and analyzed and reviewed the literature relevant to this case report. IV performed the histological examination and was a major contributor to writing the manuscript. RS was largely involved in patient management and also contributed to writing the article. RWS performed the operative resection of the tumor and critically revised the article. All authors read and approved the final manuscript.
